# Cinobufagin Is a Selective Anti-Cancer Agent against Tumors with EGFR Amplification and PTEN Deletion

**DOI:** 10.3389/fphar.2021.775602

**Published:** 2021-11-29

**Authors:** Kunyan He, Guang-Xing Wang, Li-Nan Zhao, Xiao-Fang Cui, Xian-Bin Su, Yi Shi, Tian-Pei Xie, Shang-Wei Hou, Ze-Guang Han

**Affiliations:** ^1^ Key Laboratory of Systems Biomedicine (Ministry of Education), Shanghai Center for Systems Biomedicine, Shanghai Jiao Tong University, Shanghai, China; ^2^ Key Laboratory for the Genetics of Developmental and Neuropsychiatric Disorders, Bio-X Institutes, Shanghai Jiao Tong University, Shanghai, China; ^3^ Shanghai Nature Standard Technical Services Co., Ltd., Shanghai, China; ^4^ Key Laboratory for Translational Research and Innovative Therapeutics of Gastrointestinal Oncology, Department of Anesthesiology, Hongqiao International Institute of Medicine, Shanghai Jiao Tong University School of Medicine Affiliated Tongren Hospital, Shanghai, China; ^5^ Hangzhou Innovation Institute for Systems Oncology, Hangzhou, China

**Keywords:** cinobufagin, EGFR, glioblastoma, PTEN, Chansu

## Abstract

Glioblastoma multiforme (GBM) is the most common and malignant brain tumor, and almost half of the patients carrying EGFR-driven tumor with PTEN deficiency are resistant to EGFR-targeted therapy. EGFR amplification and/or mutation is reported in various epithelial tumors. This series of studies aimed to identify a potent compound against EGFR-driven tumor. We screened a chemical library containing over 600 individual compounds purified from Traditional Chinese Medicine against GBM cells with EGFR amplification and found that cinobufagin, the major active ingredient of Chansu, inhibited the proliferation of EGFR amplified GBM cells and PTEN deficiency enhanced its anti-proliferation effects. Cinobufagin also strongly inhibited the proliferation of carcinoma cell lines with wild-type or mutant EGFR expression. In contrast, the compound only weakly inhibited the proliferation of cancer cells with low or without EGFR expression. Cinobufagin blocked EGFR phosphorylation and its downstream signaling, which additionally induced apoptosis and cytotoxicity in EGFR amplified cancer cells. *In vivo*, cinobufagin blocked EGFR signaling, inhibited cell proliferation, and elicited apoptosis, thereby suppressing tumor growth in both subcutaneous and intracranial U87MG-EGFR xenograft mouse models and increasing the median survival of nude mice bearing intracranial U87MG-EGFR tumors. Cinobufagin is a potential therapeutic agent for treating malignant glioma and other human cancers expressing EGFR.

## Introduction

Epidermal growth factor receptor (EGFR) amplification and/or mutation exist in different types of cancer and triggers higher interest in EGFR as a cancer therapeutic target. Therefore, the EGFR target therapy has been applied in various epithelial tumors such as lung cancer (Lynch et al., 2004, Hirsch et al., 2008), colorectal cancer (Gibson et al., 2006), and head and neck cancer (Sundvall et al., 2010). Glioblastoma multiforme (GBM) is the most common and malignant brain tumor in adults, with overall survival of 15–16 months and a 5-year survival rate of 5% (Ostrom et al., 2015). Standard of care is surgery followed by radio-chemotherapy and adjuvant chemotherapy (Touat et al., 2017). EGFR amplification, deletion, point mutations, and/or translocation are reported in more than half of glioblastomas (Libermann et al., 1985; Brennan et al., 2013; Eskilsson et al., 2018). GBM still remains a challenge because these tumors are resistant to anti-EGFR therapy, always relapse (Stupp et al., 2009) and recurrent tumors are less sensitive to chemotherapy than the primary tumor, developing novel anti-EGFR agents remains urgent (Campos et al., 2016; Eskilsson et al., 2018). Almost half of the patients carry EGFR-driven tumor with phosphatase and tensin homolog (PTEN) deletion, which are resistant to EGFR target therapy, implying PTEN deficiency plays an important role in resistance to anti-EGFR therapy (Mellinghoff et al., 2007; Arif et al., 2018; Brito et al., 2019). Using PTEN-deficient glioblastoma cell line U87MG-EGFRvIII screening and U87MG-PTEN counter-screening, we have previously reported that G5-7 selectively blocked Janus kinase 2 (Jak2), preventing GBM proliferation (He et al., 2013).

Chansu is extracted from *Bufo bufo gargarizans* Cantor and *Bufo melanostictus* Schneider families, which has been widely used to treat swelling, pain, and heart failure for thousands of years in China (Qi et al., 2018). Cinobufacini (Huachansu), an intravenous formulated extract from toads, has been applied to treat different types of malignant cancers and hepatitis B virus infection (Meng et al., 2009; Cui et al., 2010; Meng et al., 2012; Zhang et al., 2018; Li et al., 2020; Zhang et al., 2020). Cinobufagin, the major bioactive component of Chansu and Huachansu, can inhibit tumor growth through decreasing oncogene expression, blocking the cell cycle, triggering apoptosis, decreasing the new blood vessel formation, enhancing immune response, etc. (Wang et al., 2010; Qi et al., 2011a; Yin et al., 2013; Li et al., 2015; Zhang et al., 2016; Cao et al., 2017; Dai et al., 2018; Zhu et al., 2018; Li et al., 2019; Pan et al., 2019). Cinobufagin’s anti-cancer effects have garnered considerable attention in lung cancer, colorectal cancer, liver cancer, and gastric cancer (Qi et al., 2011b; Zhang et al., 2016; Lu et al., 2017; Xiong et al., 2019). Recently, cinobufagin combined with thalidomide was used to treat patients with lung cancer cachexia in a clinical study (Xie et al., 2018). Nonetheless, which types of the malignant tumors are sensitive to cinobufagin and the mechanisms of action remain unknown.

Here, we screened the library from Traditional Chinese Medicine for individual compounds against GBM cells with EGFR amplification and PTEN deletion and successfully identified cinobufagin, the major active ingredient of Chansu. We found that Cinobufagin specifically blocked proliferation of cancer cells with EGFR expression and PTEN deletion enhanced its anti-cell proliferation effects. *In vivo* study showed that cinobufagin suppressed the most malignant glioblastoma, U87MG-EGFR, xenograft tumor growth and extended the life span of nude mice. Cinobufagin might be a therapeutic compound for the treatment of malignant glioma and other human cancers expressing EGFR.

## Materials and Methods

### Cells, Reagents, and Mice

Human glioblastoma cell lines were gifts from Keqiang Ye at Emory University. Liver cancer cell lines (except MHCC97H) were purchased from ATCC. MHCC97H, lung cancer, and colorectal cell lines were from a national collection of authenticated cell cultures of China. All cells were maintained in DMEM or RPMI1640 with 10% FBS and 1× penicillin/streptomycin/glutamine. U87MG was stably transfected with vector control, PTEN, epidermal growth factor receptor variant III (EGFR vIII), and EGFR, and the stable transfected cells were maintained with various antibiotics: 400 μg/ml of G418 for PTEN, 0.7 μg/ml of puromycin for wild-type EGFR, and 150 μg/ml of hygromycin for EGFRvIII. LN229-EGFR and SF763-EGFR stable transfected cells were maintained with 150 μg/ml of hygromycin. All experiments were performed with mycoplasma-free cells. The primary antibodies were from Cell Signaling and the second antibodies were from Invitrogen. The Traditional Chinese Medicine monomer library composed of over 600 natural compounds was provided by Nature Standard of Shanghai. CytoTox 96 Non-Radioactive Cytotoxicity Assay and Caspase-Glo 3/7 Assay were from Promega. Male nude mice (BALB/c nu/nu, 6 weeks of age) and male C57BL/J (MGI Cat# 2160531, 8 weeks of age) mice were obtained from Shanghai SLAC Laboratory Animal Co. Ltd. Mice were housed with a maximum of five per cage and fed autoclaved chow and water with 12 h light and dark cycles. All efforts were made to minimize discomfort to the animals. The animals required physical restraint for injection of tumor cells and delivery of drugs, and measurement of tumor size with a caliper (hand-held). All procedures were approved by the Institutional Animal Care and Use Committee of Shanghai Jiao Tong University and were performed at the Animal Center of Shanghai Jiao Tong University.

### *In Vitro* Proliferation, Cytotoxicity, and Apoptosis Assay

Three thousand cells were seeded in each well of a 96-well plate. The next day, the medium was replaced with fresh medium containing different concentrations of compounds or vehicle. Cells were incubated at 37°C for the indicated times. The cell proliferation, released lactate dehydrogenase (LDH), and caspase 3/7 activity were monitored by cell counting kit 8 (CCK8), CytoTox 96 Non-Radioactive Cytotoxicity, and Caspase-Glo 3/7 assays, respectively, according to the manufacturer’s protocols.

### Subcutaneous and Intracranial Xenograft Model

Cells (2×10^6^) in phosphate buffered saline (PBS) were inoculated subcutaneously into 6-week-old male nude mice. Treatment commenced once tumors had reached a mean volume of 100 mm^3^. The mice were randomly divided into three groups and were treated with vehicle control or cinobufagin (1 and 5 mg kg^−1^) by daily intraperitoneal injection for indicated days. Tumor volume was calculated using the formula (length × width^2^)/2, where length is the longest axis and width is the measurement at right angles to the length. For the intracranial model, after anesthetized by intraperitoneal injection of mixture of ketamine/xylazine (95:5 mg/kg), mice were placed in the stereotaxic instrument and cells (1×10^5^) were stereotactically inoculated into the right striatum, 3 mm below the dural surface on day 0. On day 7, the mice were intraperitoneally injected with D-luciferin potassium salt and were examined with IVIS Lumina II to confirm tumor formation. Then, the mice were randomly divided into two groups and were daily intraperitoneally injected with vehicle or cinobufagin (5 mg kg^−1^). Ten days after drug treatment, mice from each group were analyzed by IVIS Lumina II again for tumor luminescence intensity.

### Flow Cytometric Analysis

Cells were treated with vehicle or cinobufagin for 24 h, then harvested and washed twice with cold PBS. After fixed in 70% ethanol at −20°C for at least 24 h, the cells were washed with PBS and incubated with propidium iodide (20 μg/ml)/RNase A (20 μg/ml) in PBS for 45 min. The samples were analyzed on a BD&LSR Fortessa.

### Immunofluorescence Staining

For immunofluorescence staining, paraffin-embedded tissue sections from intracranial model were deparaffinized in xylene, rehydrated in graded alcohols, and were boiled in 10 mM sodium citrate buffer (pH 6.0) for 10 min. Frozen sections from subcutaneous model were rehydrated in PBS. Then, the sections from both models were permeabilized with PBS+0.1% Triton X-100. The sections were blocked with 1% bovine serum albumin in PBS at 37°C for 30 min followed primary anti-Ki67 (1:300) or anti-active caspase 3 (1:400) incubation at 4°C overnight. The sections were washed with PBS and incubated with Alexa Fluor 488–labeled goat anti-mouse or anti-rabbit IgG antibody (1:500) at room temperature for 60 min, followed by rinsing with PBS for 10 min and staining with 4′,6-diamidino-2-phenylindole (DAPI) for another 10 min at room temperature. After mounting, the sections were examined under a fluorescence microscope.

### Statistics Analysis

Data are presented as means ± SD. Statistical evaluation was carried out by Student’s t-test (two groups) or ANOVA (three groups) plus Bonferroni *post hoc*. Data were considered statistically significant when *p* < 0.05.

## Results

### Screening Reveals Cinobufagin That Selectively Inhibits Proliferation of EGFR-Amplified, PTEN-Deficient Glioblastoma Cells

**FIGURE 1:**
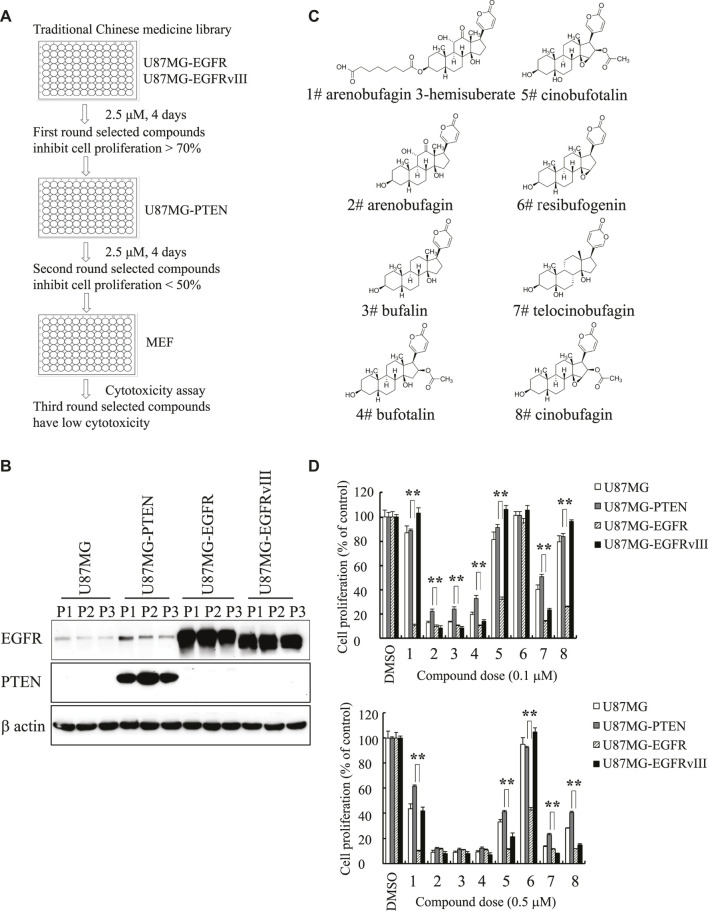
Identification of cinobufagin and its SAR study. **(A)** The diagram of drug screening strategy. **(B)** Expression check of EGFR and PTEN in stable cell lines U87MG-control, U87MG-PTEN, U87MG-EGFR, and U87MG-EGFRvIII from the indicated passages. **(C)** Chemical structures of 8 monomers of Chansu. **(D)** SAR study of Chansu’s monomers from **(C)** and their antiproliferative effects on U87MG-control, U87MG-PTEN, U87MG-EGFR, and U87MG-EGFRvIII cells. 1 to 8 in horizontal axis represent the 8 compounds at panel **(C)**. Data are means ± SD.

To seek compounds effective in treating the most malignant type of GBM with EGFR amplification and PTEN deletion, we developed a cell-based screening assay to identify individual compounds from Traditional Chinese Medicine that specifically block the cell proliferation of U87MG-EGFR with EGFR amplification and PTEN deficiency but mildly inhibit the proliferation of U87MG-PTEN cells without EGFR amplification and with wild-type PTEN. The positive compounds that markedly inhibited the proliferation of U87MG-EGFR cells over 50% were applied to the second-round screening in U87MG-PTEN cells. The compounds that exhibited a robust antiproliferative effect on U87MG-EGFR cells but a weak effect on U87MG-PTEN cells were chosen for further evaluating the cytotoxicity toward mouse embryonic fibroblasts (MEFs). The promising compounds that produced cytotoxicity less than 20% in MEFs were further evaluated. After screening approximately 600 individual compounds of Chinese medicine by this process, we identified two compounds, cinobufagin and resibufogenin, that selectively inhibit U87MG-EGFR not U87MG-PTEN cell proliferation. Interestingly, both cinobufagin and resibufogenin are extracted from Chansu with similar chemical backbones (Figure 1).

To search for more effective and selective derivatives, we collected the bioactive compounds of Chansu with similar structure and performed structure–activity relationship (SAR) studies, and found that arenobufagin 3-hemisuberate, cinobufotalin, and cinobufagin at 0.1 μM exhibited more potent antiproliferative effect on U87MG-EGFR cells and almost no effects on other cells derived from the same parent cells, including U87MG-Vehicle, U87MG-PTEN, and U87MG-EGFRvIII. One of the derivatives, resibufogenin, which is another positive hit identified in the screening assay, also exhibited selective antiproliferative activity against U87MG-EGFR at 0.5 μM but no effect at 0.1 μM. Interestingly, cinobufagin and cinobufotalin exhibited potent antiproliferative activity against U87MG-EGFR at both 0.1 and 0.5 μM. They also displayed effective antiproliferative effect on U87MG-EGFRvIII at 0.5 μM, which is often overexpressed in GBM (Figure 1D). It is worth noting that cinobufagin, resibufogenin, and cinobufotalin have similar chemical structures except for the acetyl group and hydroxyl groups. Cinobufagin with acetylation was more potent than resibufogenin, implying the acetyl group is critical for its biological effect. Cinobufotalin with an additional 1-hydroxy group was a little bit weak than cinobufagin. So, we selected cinobufagin for further biochemical and pharmacological studies.

### Cinobufagin Inhibits EGFR and its Downstream Signaling Cascades, Induces Apoptosis and Cytotoxicity of EGFR Amplified Cells

**FIGURE 2:**
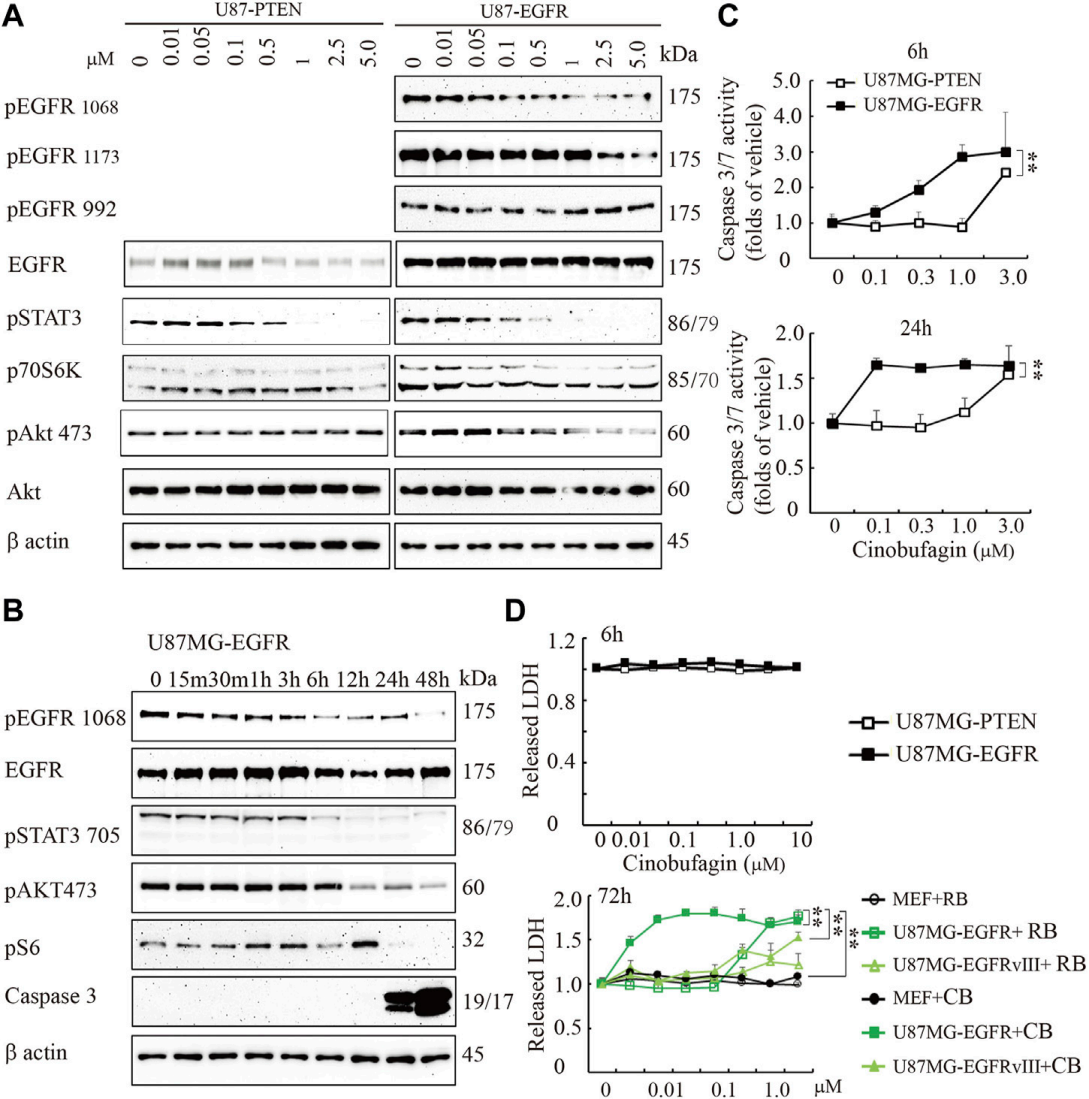
Cinobufagin blocks EGFR phosphorylation and induces cell apoptosis and cytotoxicity. **(A)** Cinobufagin blocks EGFR signaling in a dose-dependent manner. Western blot analysis of U87MG-EGFR and U87MG-PTEN cells treated with different concentrations of cinobufagin for 6 h. Blots are representative of three experiments. **(B)** Cinobufagin blocks EGFR signaling in a time-dependent manner. Western blot analysis of U87MG-EGFR cells treated with 0.5 μM cinobufagin for different times as indicated. Blots are representative of three experiments. **(C)** Cinobufagin specifically induces apoptosis in U87MG-EGFR cells. The cells treated with a range of doses of cinobufagin for 6 or 24 h, then the caspase 3/7 activity was measured with Caspase-Glo 3/7 Assay. **(D)** Cinobufagin specifically induces cytotoxicity of U87MG-EGFR cells. The cells were treated with different doses of cinobufagin (CB) or resibufogenin (RB) for 6 or 72 h, then the released LDH were measured with cytoTox 96 Non-radioactive Cytotoxicity Assay. Data are means ± SD.

To explore the potential molecular mechanisms of cinobufagin inhibiting U87MG-EGFR cell proliferation, we conducted the titration assay followed by Western blotting to monitor the signaling pathways mediated by EGFR. Immunoblotting showed that phosphorylation of EGFR at both Tyr1068 and Tyr1173 was inhibited by cinobufagin in U87MG-EGFR cells, which was almost undetectable in U87MG-PTEN cells (Figure 2A). Signal transducer and activator of transcription 3 (STAT3), another promising therapeutic target for GBM patients, is also a critical mediator of EGFR. Inhibition of STAT3 enhances the efficiency of EGFR inhibitor in PTEN-deficient and PTEN-intact GBM cells (Zulkifli et al., 2017). As expected, the phosphorylation of STAT3 was dramatically decreased by cinobufagin in U87MG-EGFR and U87MG-PTEN cells. Consistent with the decreased phosphorylation of EGFR and STAT3, the phosphorylation of downstream molecular, V-akt murine thymoma viral oncogene homolog (Akt), was also decreased by cinobufagin in U87MG-EGFR cells, not in U87MG-PTEN cells (Figure 2A). The time-course assay demonstrated that cinobufagin initially blocked EGFR phosphorylation in U87MG-EGFR cells at 15 min, and obviously decreased EGFR and STAT3 phosphorylation from 6 to 48 h (Figure 2B). Flow cytometric analysis revealed that cinobufagin substantially arrested cell cycle at G2 and S phases in U87MG-Vehicle, U87MG-EGFRvIII, and U87MG-EGFR cells, except for U87MG-PTEN cells, suggesting that the cell cycle arrest contributes to cinobufagin’s antiproliferative effect in GBM cells (Supplementary Figure S1).

The time-course assay also showed that cinobufagin at 0.5 μM strongly increased active caspase 3 at 24 and 48 h, detected by Western blotting. The more sensitive luminescence assay for caspase 3/7 activity demonstrated that cinobufagin markedly induced apoptosis of U87MG-EGFR cells at 0.1 μM and reached the highest rate at 1 μM but did not affect U87MG-PTEN cells at 1 μM after 6 h of treatment. When incubation time was extended to 24 h, cinobufagin at 0.1 μM induced the highest apoptosis rate in U87MG-EGFR and did not affect U87MG-PTEN cells except at 1 μM (Figure 2B,C). We treated the cells with different doses of cinobufagin for 6 or 72 h. The cytotoxicity assay showed that even 10 μM cinobufagin did not induce cytotoxicity in both U87MG-EGFR and U87MG-PTEN cells after 6 h of incubation. If the incubation time was extended to 72 h, cinobufagin significantly induced stronger cytotoxicity in U87MG-EGFR cells in a dose-dependent manner than that in U87MG-EGFRvIII cells or MEFs, while its derivative compound resibufogenin induced weak cytotoxicity (Figure 2D).

To study whether cinobufagin exerts its anti-cell proliferative effect directly through inhibiting EGFR kinase activity, we performed an *in vitro* kinase assay using the commercial EGFR protein in the presence of different doses of cinobufagin and found that even the high dose of cinobufagin did not affect EGFR kinase activity, while the EGFR inhibitor, erlotinib, robustly blocked the EGFR kinase activity (Supplementary Figure S2). The kinase activity profiling and phosphatase activity profiling showed that none of the 83 kinases and 5 phosphatases tested was affected by cinobufagin (Supplementary Figure S3, Supplementary Tables S1, S2).

### Cinobufagin Inhibits Proliferation of EGFR-Expressing Cancer Cells

**FIGURE 3:**
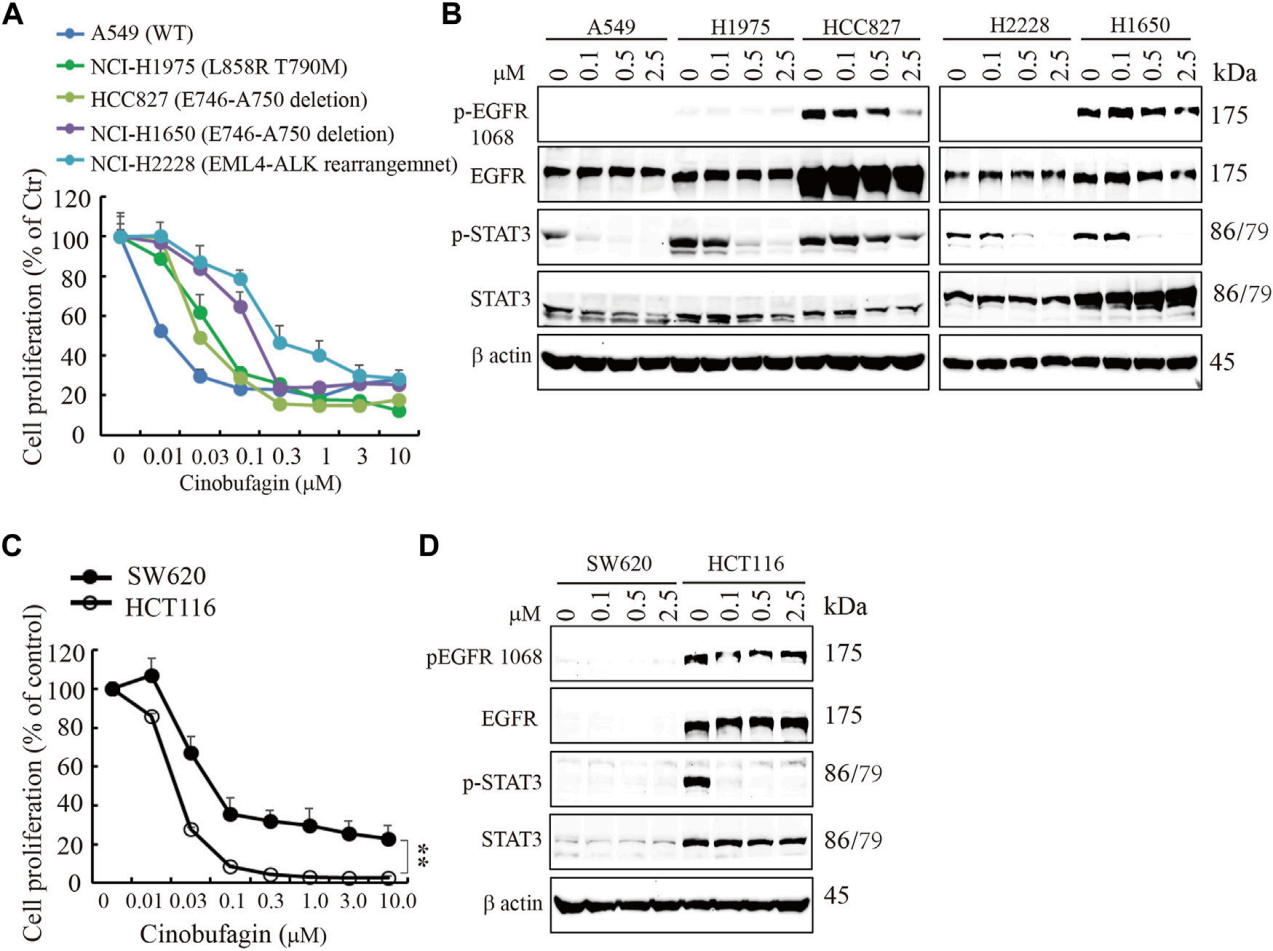
Cinobufagin inhibits proliferation of EGFR expression cancer cells. **(A)** Cinobufagin inhibits proliferation of non–small lung cancer cell lines with EGFR expression. The non–small lung cancer cell lines with different status of EGFR were treated with a range of doses of cinobufagin for 72 h, followed by CCK8 assay. **(B)** EGFR expression and its downstream signaling analysis. The non–small lung cancer cell lines were treated with indicated doses of cinobufagin for 6 h, followed by Western blot analysis of EGFR and its downstream molecules. **(C)** Cinobufagin inhibits proliferation of colorectal cells with EGFR expression. Colorectal cells were treated with cinobufagin for 72 h, then followed by CCK8 assay. **(D)** EGFR expression and its downstream signaling analysis by Western blot. Colorectal cells were treated with indicated doses of cinobufagin for 6 h, followed by Western blot analysis of EGFR and its downstream molecules. Data are means ± SD.

EGFR is often mutated and/or amplified in various human cancers and is an important target for multiple cancer therapies in the clinical practice (Yarden and Pines, 2012). To characterize whether EGFR-overexpressed or mutated carcinoma cells are sensitive to cinobufagin, we screened more cancer cell lines, including glioblastoma, lung cancer, colorectal cancer (CRC), and liver cancer cell lines, with different status of EGFR. The glioblastoma cell lines without EGFR amplification were less sensitive to cinobufagin compared with the EGFR-overexpressed GBM cells (Supplementary Figure S4A). Interestingly, cinobufagin strongly inhibited the proliferation of lung cancer cells with wild-type or mutated EGFR expression. A549 with wild-type EGFR was most sensitive to cinobufagin among these selected lung cancer cells (Figure 3A,B). We included two colorectal cancer cell lines to test their sensitivities to cinobufagin. The cell proliferation assays showed that HCT-116 with high EGFR expression was more sensitive to cinobufagin compared with the SW620 with undetectable EGFR (Figure 3C,D). We also chose different liver cancer cell lines to test whether their sensitivities to cinobufagin are related to EGFR status. Noticeably, the most sensitive SK-HEP-1 cells expressed high EGFR, while the resistant HepG2 cells expressed undetectable EGFR (Supplementary Figure S4B,C). Hence, cinobufagin exerts selective anti-cancer effect toward EGFR-overexpressed cancer cells.

### PTEN Deficiency Enhances Cinobufagin’s Antiproliferation Effect

**FIGURE 4:**
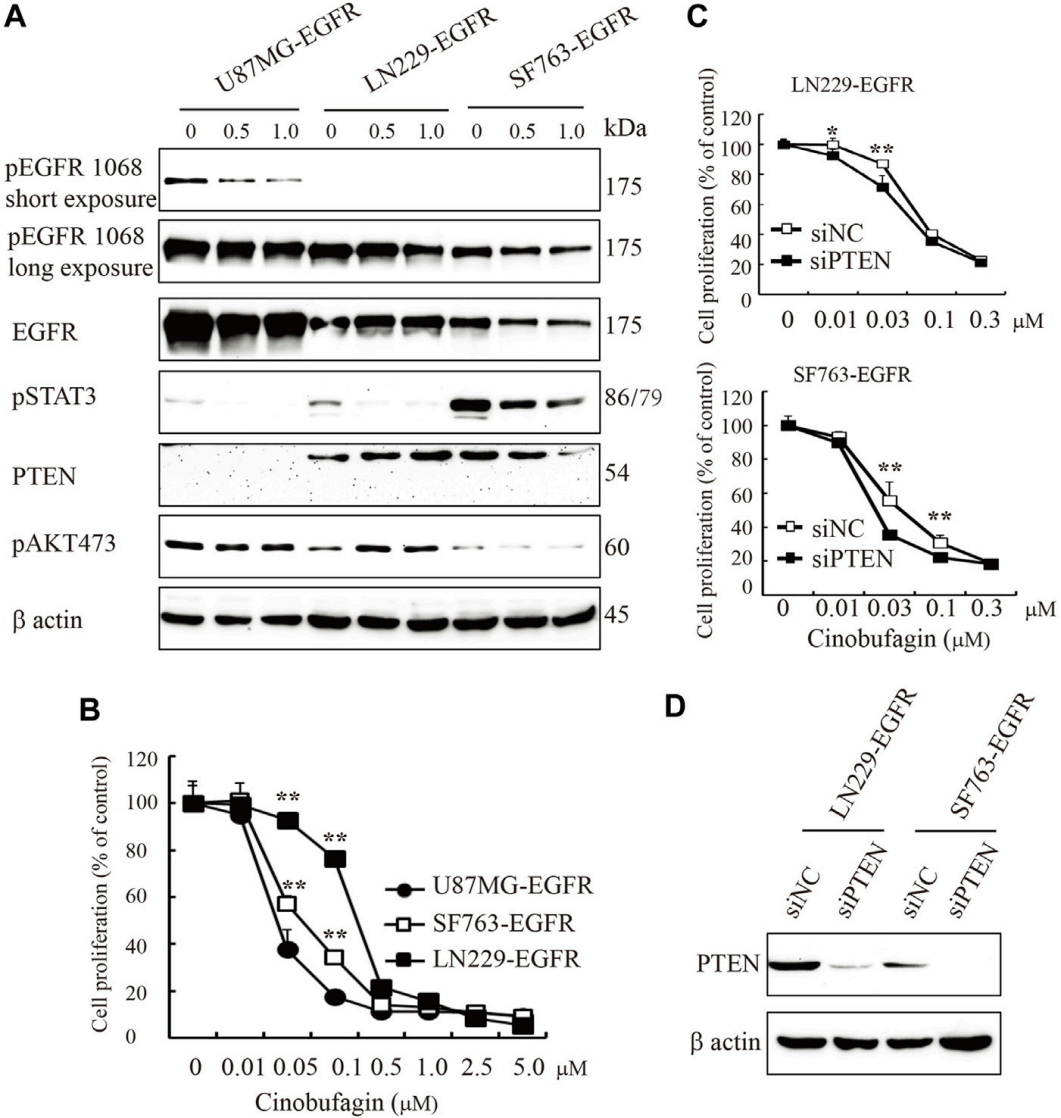
PTEN deficiency enhances cinobufagin’s antiproliferation effect. **(A)** PTEN, EGFR expression, and its downstream signaling molecules analyzed by Western blot. U87MG-EGFR, LN229-EGFR, and SF763-EGFR cells were treated with cinobufagin for 6 h, followed by Western blot analysis of PTEN, EGRR, and its downstream molecules. **(B)** Cinobufagin more efficiently inhibits cell proliferation of glioblastoma cell lines with EGFR overexpression and PTEN deficiency. Glioblastoma cells were treated with a range of doses of cinobufagin for 72 h, then followed by CCK8 assay. **(C)** Proliferation of LN229-EGFR and SF763-EGFR cells transfected with control or PTEN siRNA for up to 4 days, followed by CCK8 assay. **(D)** Western blot checks the PTEN expression. Data are means ± SD.

EGFR amplification accompanied by loss of PTEN is common in clinical trials; these patients carrying EGFR-driven tumors with PTEN mutation are resistant to anti-EGFR treatment. In the initial screening, we selected the compounds that specifically blocked the most malignant GBM cells’ (U87MG-EGFR/PTEN null) proliferation and displayed less or no effects on PTEN normal cells. As expected, we identified cinobufagin as the leading compound, which efficiently blocked U87MG-EGFR cell proliferation and had less effect on U87MG-PTEN cells. We tested the cinobufagin antiproliferation effect on other GBM cells LN229-EGFR and SF763-EGFR, which overexpress EGFR and have normal PTEN, and observed that cinobufagin had less effect on LN229-EGFR and SF763-EGFR than that on PTEN-deficient U87MG-EGFR. To confirm PTEN’s role, we further knocked down PTEN at both LN229-EGFR and SF763-EGFR and found that these cells with low PTEN were more sensitive to cinobufagin (Figure 4).

### Cinobufagin Inhibits Subcutaneous Xenograft Growth in Nude Mice

**FIGURE 5:**
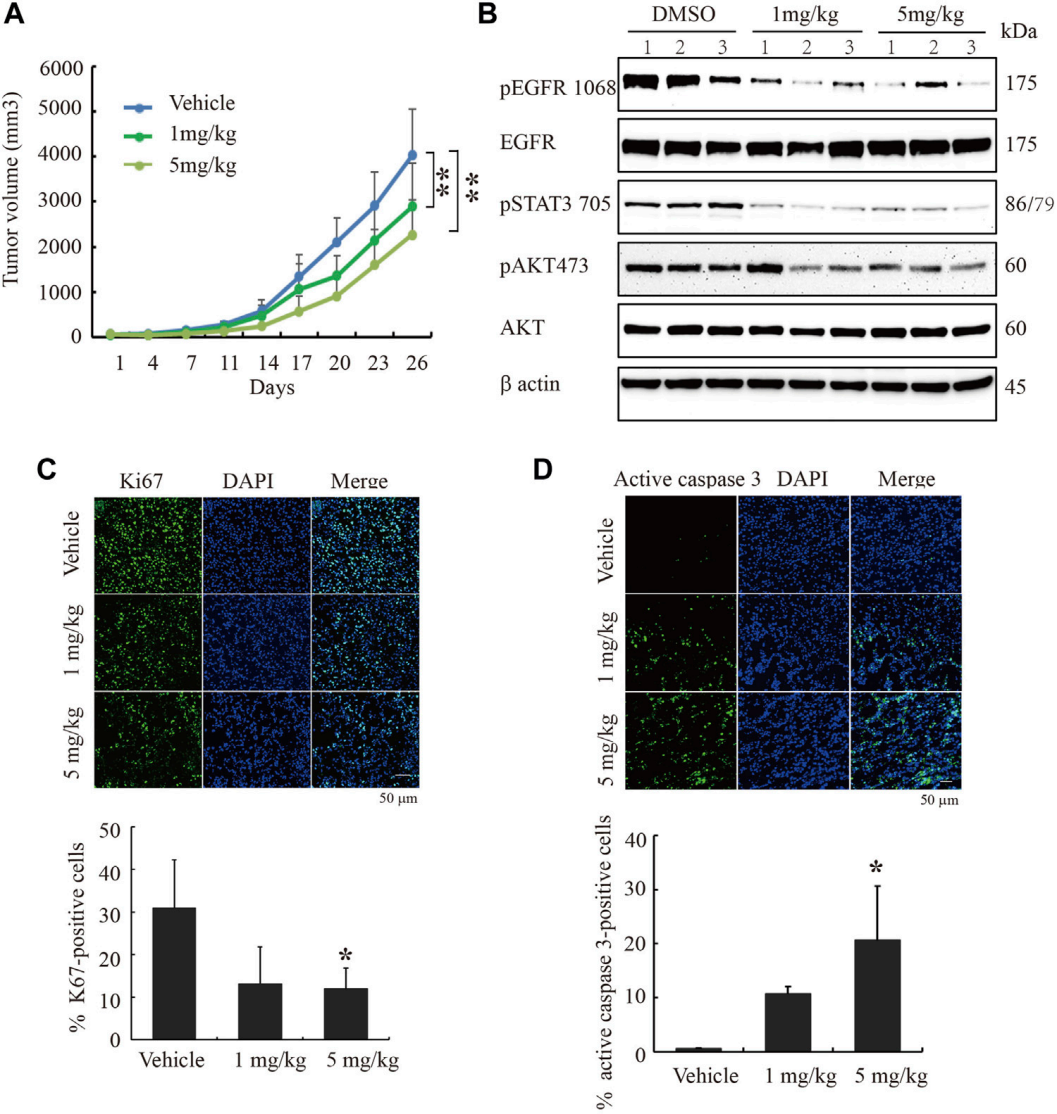
Cinobufagin blocks subcutaneous tumor growth of U87MG-EGFR cells. **(A)** Cinobufagin significantly decreases the tumor volumes in subcutaneous U87MG-EGFR xenograft model. U87MG-EGFR cells were subcutaneously inoculated into nude mice, and after the tumors formed, the nude mice were intraperitoneally injected with vehicle (0.5% cyclodextrin) or cinobufagin at doses of 1 and 5 mg kg^−1^, respectively. Data represent means ± SD (*n* = 6/group). **(B)** Cinobufagin inhibits EGFR and STAT3 signaling in subcutaneous tumors. The tumor tissue lysates from vehicle- or drug-treated samples were analyzed by Western blotting with the indicated antibodies. **(C,D)** Cinobufagin inhibits cell proliferation and induces apoptosis in subcutaneous tumors. The immunofluorescence staining of Ki67 and active caspase 3 on tumor sections derived from animals treated with or without cinobufagin. Quantification of Ki67 and active caspase 3–positive cells in subcutaneous tumor. Data represent means ± SD. *p < 0.05; **p < 0.01.

To study cinobufagin’s anti-cancer effect *in vivo*, the U87MG-EGFR cells were subcutaneously inoculated in nude mice. After daily intraperitoneal injection of vehicle or drugs (1 or 5 mg kg^−1^) for 26 days, tumor growth in the cinobufagin-treated group was much slower than that in the vehicle-treated group (Figure 5A). To explore the molecular mechanisms of cinobufagin *in vivo*, we monitored the major signaling effectors of EGFR and STAT3 in the tumors by Western blotting, and found that *p-*EGFR and its downstream signaling machinery *p*-STAT3 and *p*-Akt were obviously decreased by cinobufagin (Figure 5B). The cell proliferation marker (Ki67) staining was also significantly decreased in tumors treated with cinobufagin compared with that in vehicle-treated tumors (Figure 5C), suggesting that this compound strongly inhibits tumor cell proliferation *in vivo*. To explore whether this compound also affects tumor growth via triggering apoptosis, the active caspase-3 immunostaining was conducted, and the data showed that the cinobufagin triggered stronger apoptosis, whereas vehicle elicited negligible apoptosis (Figure 5D). These data indicated that cinobufagin blocks tumor growth *in vivo* through inhibiting cell proliferation and triggering apoptosis. Thus, cinobufagin inhibits EGFR and STAT3 signaling to block tumor growth in nude mice.

### Cinobufagin Decreases Intracranial Tumor Growth and Extends the Life Span of Tumor-Bearing Nude Mice

**FIGURE 6:**
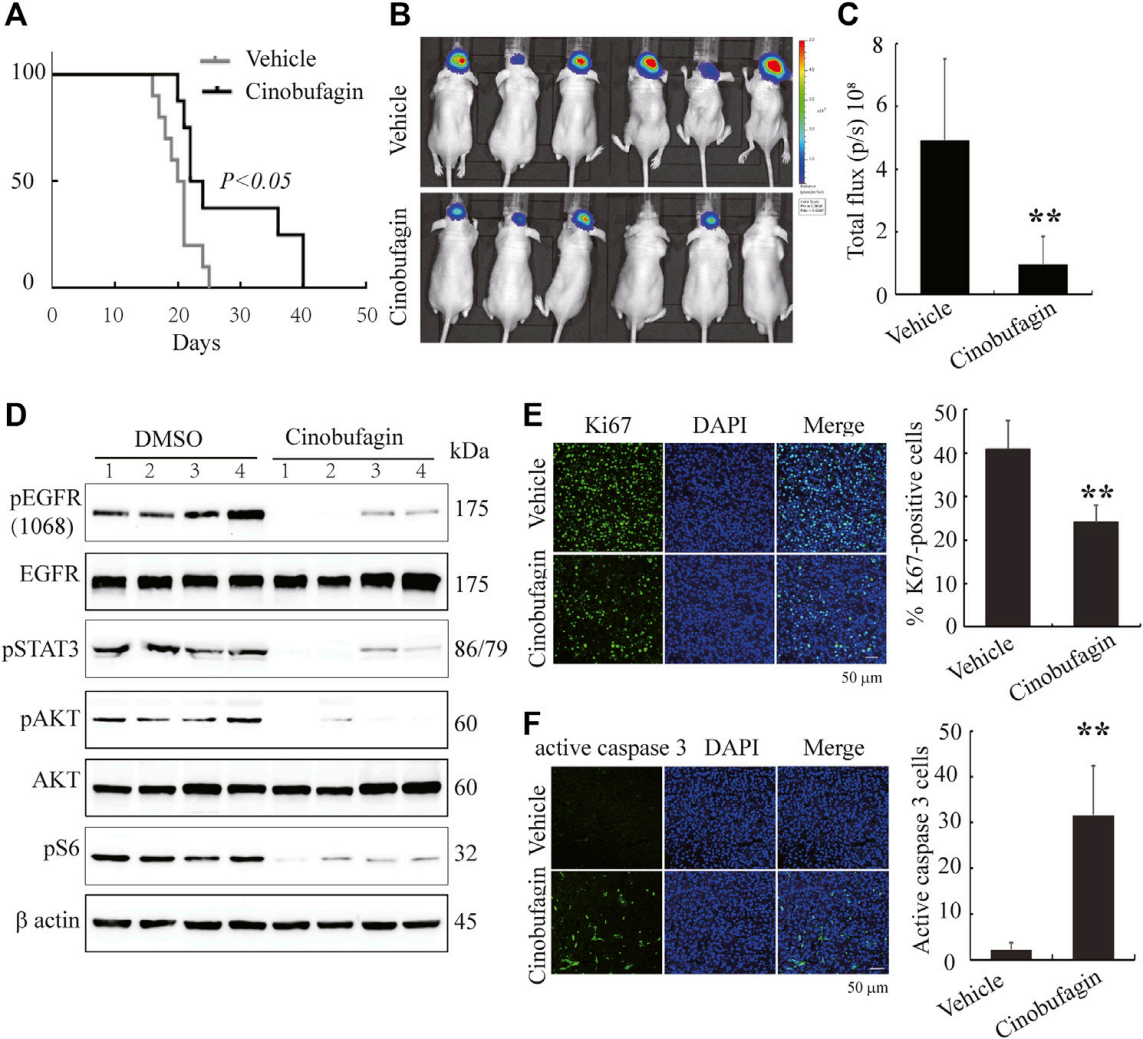
Cinobufagin suppresses tumor growth of U87MG-EGFR cells and elongates nude mice life span. **(A)** Survival curves of brain tumor–bearing mice. After confirmation of brain tumor formation by luminance imaging, mice were intraperitoneally injected with vehicle or cinobufagin once a day until the termination of the experiment (*n* = 8 for vehicle group or 10 for cinobufagin group). The cinobufagin treatment group (23 days) showed a significant improvement in their survival compared with the vehicle treatment group (20.5 days, *p* < 0.05). **(B)** Luminance imaging of individual mice 10 days after cinobufagin treatment. The presence of a glioma is detected through the colorful luminance intensity. **(C)** Quantitative analysis of intracranial tumor luminance intensity in mice treated with vehicle or cinobufagin. Cinobufagin significantly suppressed tumor growth compared with the vehicle. Data represent means ± SD (*p < 0.05, *n* = 6). **(D)** Cinobufagin inhibits EGFR and STAT3 signaling in intracranial tumor. The tumor tissue lysates from vehicle or drug-treated mice were analyzed by immunoblotting with the indicated antibodies. **(E,F)** Cinobufagin inhibits cell proliferation and induces apoptosis in intracranial tumor. The immunofluorescence staining of Ki67 **(E)** and active caspase 3 **(F)** on tumor sections derived from animals treated with or without cinobufagin. Quantification of Ki67 and active caspase 3–positive cells in intracranial tumor. Data represent means ± SD. *p < 0.05; **p < 0.01 compared with the vehicle group.

To further evaluate the therapeutic potential of cinobufagin on the orthotopic glioblastomas, we stereotactically inoculated U87MG-EGFR cells in the corpus striatum of nude mice and monitored tumor growth by luciferase bioluminescence imaging. After confirming tumor formation in the brains on day 7, each group of animals was intraperitoneally injected with vehicle (0.5% β-cyclodextrin) or cinobufagin (5 mg kg^−1^) once a day. The survival was evaluated by Kaplan–Meier analysis. The median survival time (23 days) for the cinobufagin treatment group was significantly longer than 20.5 days for the vehicle group (Figure 6A). The mice were subjected to luciferase bioluminescence imaging 10 days after treatment. The luminescence intensity of brain tumor treated with cinobufagin was decreased about 70% (Figure 6B,C).

Next, we tested the signaling molecules in these samples and found that cinobufagin strongly decreased *p-*EGFR, *p-*STAT3, and *p-*Akt levels in the intracranial tumors as compared with the vehicles (Figure 6D), which were consistent with what were observed in subcutaneous tumors. Moreover, Ki67 and active caspase-3 immunostaining of intracranial tumors were significantly decreased in cinobufagin-treated group compared with those in the vehicle-treated group (Figure 6E,F). Therefore, cinobufagin blocks EGFR/STAT3 signaling in glioblastoma and shrinks brain tumors, elongating nude animal survival.

## Discussion

We found cinobufagin, a monomer of Chansu from Traditional Chinese Medicine, that demonstrated selective antiproliferative activity against EGFR-overexpressed and PTEN-deficient GBM cells, compared with normal EGFR and PTEN cells. It also exhibited antiproliferative activity in various carcinoma cancer cells with EGFR expression, including lung cancer cells, colorectal cancer cells, and liver cancer cells. Cinobufagin blocked the oncogenic pathways mediated by EGFR and STAT3, and triggered apoptosis in a dose-dependent manner *in vitro* and *in vivo* (Figures 2, 5, 6). Intraperitoneal administration of cinobufagin dose-dependently inhibited the tumor growth of U87MG-EGFR in subcutaneous xenograft model. Moreover, cinobufagin significantly inhibited tumor growth and extended the life span of intracranial xenograft mice, implying that this monomer itself or its metabolites could cross the brain–blood barrier to exert anti-tumor effects (Figure 6).

The early studies showed that the anti-cancer activity of cinobufagin is manifested in eliciting apoptosis, inhibiting autophagy, blocking cell cycles, and inhibiting cell proliferation (Zhang et al., 2016; Zhu et al., 2018; Dai et al., 2018; Cao et al., 2017; Zhang et al., 2019). In the study, we demonstrated that cinobufagin exhibited specific antiproliferation effect in the special carcinoma cells with EGFR expression, including glioblastoma cells, lung cancer cells, colorectal cells, and hepatocellular carcinoma cells (Figures 1A, 3A,C, 4B,C, Supplementary Figure S4A,B). We further characterized that cinobufagin displayed antiproliferation effects on the cells with EGFR mutation, which are resistant to EGFR inhibitor (Figure 3A). Zhang et al. and other groups’ reports indicate that cinobufagin blocks Akt signaling pathway, decreases Bcl-2, and induces mitochondrial cytochrome *c* release to trigger apoptosis, or inhibits STAT3 and Notch pathways to suppress tumor cell growth (Yin et al., 2013; Zhang et al., 2016; Zhang et al., 2019). Pan et al. reported that cinobufagin arrests cell cycle at G2/M or S phase and induces apoptosis in melanoma and nasopharyngeal carcinoma cells (Pan et al., 2019; Pan et al., 2020). Our work is consistent with these studies and further demonstrated that cinobufagin inhibits EGFR/STAT3 and its downstream signaling to arrest cell cycle at G2/M phase, to inhibit cell proliferation, and to promote apoptosis (Figures 2A,B, 3B,D). Our work also validated that cinobufagin inhibited tumor growth and elongated nude mice life span via blocking EGFR and STAT3 signaling pathways and inducing apoptosis *in vivo* (Figures 5B–D, 6D–F). Our study demonstrates that the malignant tumors with wild-type or mutated EGFR are sensitive to cinobufagin. Unfortunately, the *in vitro* kinase assay displayed that cinobufagin did not inhibit EGFR and STAT3 activity (Supplementary Figure S2 and Supplementary Table S1). It also cannot affect the EGFR upstream kinase Jak2 or phosphatases activity (Supplementary Figure S3, Supplementary Tables S1, S2). We failed to identify the cellular target of cinobufagin after screening around 90 kinases and phosphatases (Supplementary Tables S1, S2).

Chansu, a traditional medicine from Chinese toad, has been widely used in clinic in Asian countries. Huachansu, which is a water-soluble extract from toad and an intravenous formulated agent, has been used in clinic for treating late-stage liver cancer, lung cancer, and gastric cancer (Gibson et al., 2006; Meng et al., 2009; Zhang et al., 2018; XU and WU, 2017; MA and LU, 2011). Both Chansu and Huachansu are mixtures, which include various bioactive components, such as cinobufagin, bufotalin, bufalin, arenobufagin, and so on, and show potential anti-cancer effect, as well as side effects (Meng et al., 2016). In the current study, we collected the bioactive components of Chansu and performed SAR analysis, and found that each derivative had unique selectivity and antiproliferation efficacy. Among them, cinobufagin, cinobufotalin, and resibufogenin have similar chemical structure except for the acetyl group and hydroxy groups. Cinobufagin and cinobufotalin, having same acetylation and different hydroxy, exhibit better selectivity and higher efficiency against cancer cells with EGFR expression and PTEN deletion than resibufogenin (Figure 1D), implying the acetyl group increases the inhibitory effect and 1-hydroxy is not critical. Toma et al. reported that cinobufagin can be metabolized to desacetylcinobufagin (Toma et al., 1987), and our study showed that resibufogenin without the acetyl group had less inhibitory effect, implying that the deacetylation of cinobufagin might contribute to its low anti-cancer efficacy *in vivo*. In the near future, we could focus on cinobufagin and modify it to enhance its anti-cancer efficiency and selectivity *in vitro* and *in vivo*, and to avoid the side effect.

Chansu has been used in clinic for a long time, but its toxicity cannot be ignored. Two preclinical studies showed that intraperitoneal injection of 10 mg kg^−1^ cinobufagin is tolerable in nude mice (Lu et al., 2017). However, in our study, we tried the highest dose of cinobufagin (10 mg kg^−1^) via intraperitoneal injection and found that one-third of the nude mice were dead, and then reduced the highest dose to 5 mg kg^−1^. We daily injected C57BL/J mice with 5 mg kg^−1^ cinobufagin up to 1 month and performed complete blood count and did not observed any abnormity and difference between cinobufagin and vehicle-treated groups (Supplementary Table S3). We also treated the cells *in vitro* with different doses of cinobufagin and monitored the toxicity and apoptosis. The data showed that cinobufagin did not cause cellular toxicity in MEFs, but triggered significant cell apoptosis after 6 h of treatment, and caused significant cytotoxicity and apoptosis after 72 h of treatment in U87MG-EGFR cells (Figure 2C,D). Cinobufagin’s structure is similar to ouabain, one of the digitalis drugs used to treat congestive heart failure through targeting Na^+^/K^+^-ATPase, which can trigger neuron-glia necrosis at high concentration (50–100 μM) (Xiao et al., 2002). However, ouabain inhibits cancer cell proliferation and cannot cause significant changes of intracellular ration of Na^+^/K^+^ at low concentration (<100 nM) (Kometiani et al., 2005). So cinobufagin might not induce intracellular changes of ratio of Na^+^/K^+^ to trigger cytotoxicity at low concentration (<0.5 μM). However, it could be possible that cinobufagin triggers acute cytotoxicity due to high concentration at the site of injection, leading to mice death.

At this moment, the cellular targets of cinobufagin remain unclear. Future efforts are necessary to delineate the potential molecular targets for deciphering cinobufagin’s anti-cancer effects. Optimization of this compound might warrant a new drug for the treatment of malignant glioma and other human cancers expressing EGFR.

## Data Availability

The original contributions presented in the study are included in the article/Supplementary Material; further inquiries can be directed to the corresponding authors.

## Abbreviations

AKT, v-Akt murine thymoma viral oncogene homolog; CRC, colorectal cancer; EGFR, epidermal growth factor receptor; GBM, glioblastoma multiforme; PTEN, phosphatase and tensin homolog; MEFs, mouse embryonic fibroblasts; NSCLCs, non–small cell lung carcinomas; SAR, structure–activity relationship; STAT3, signal transducers and activators of transcription 3.
