# Supplementary material for: Cinobufagin Is a Selective Anti-Cancer Agent against Tumors with EGFR Amplification and PTEN Deletion
Source: Front Pharmacol. 2021 Nov 29;12:775602. doi: 10.3389/fphar.2021.775602 (PMC8672866; doi:10.3389/fphar.2021.775602)
Supplement: Supplementary file 1 [file DataSheet1.PDF]

Supplementary Information

Supplemental Figure legends

Figure S1. Cinobufagin arrests cell cycles at S and G2 phases. (A) Cinobufagin arrests the cell cycle at the S and G2 phases. U87MG, U87MG-PTEN, U87MG-EGFR, and U87MG-EGFRvIII cells were treated with cinobufagin for 24 h followed flow cytometric analysis. (B) Cell proliferation assay. Cinobufagin preferentially blocked the cell proliferation of U87MG-EGFR cells in a dose-dependent manner. Data were expressed as mean ± SD

Figure S2. Cinobufagin doesn't affect EGFR kinase activity. EGFR (4 units) was preincubated with different concentrations of cinobufagin or EGFR inhibitor erlotinib on ice for 10 min, and then was incubated with 0.3 mM ATP on PGT-coated wells for 30 min at room temperature. The phosphorylated PGT was detected by ELISA using monoclonal anti-phosphotyrosine-HRP and OPD substrate.

Figure S3. Phosphatase profiling. (A) U87MG-EGFR cell lysates were incubated with different concentrations of cinobufagin or PTP1B inhibitor 1,2-NQ at room temperature, the reactions were initiated by addition of pNPP and measured at 405 nm at 30-sec intervals for 15 min. (B) SHIP2 immunoprecipitated from U87MG-EGFR cell lysate with SHIP2 antibody was preincubated with different concentrations of cinobufagin on ice for 30 min. The reactions were initiated by addition of PI(3,4,5)P3 and then were incubated at 37 °C for 45 min. After that, the Malachite green solution was added and incubated for 30 min at room temperature followed absorbance reading at 620 nm. (C) U87MG, U87MG-PTEN, U87MG-EGFR, and U87MG-EGFRvIII cells were treated with different concentrations of SHP2 inhibitor SSG for 3 d followed MTT assays.

Figure S4. (A) Glioblastoma cell lines were treated with different concentrations of cinobufagin for 3 d followed MTT assays. (B) Hepatocellular carcinoma (HCC) cell lines were treated with different concentrations of cinobufagin for 3 d followed MTT

assays. (C) HCC cell lines were treated with different concentrations of cinobufagin for 6 h followed Western blotting with indicated antibody.

Figure S1

A

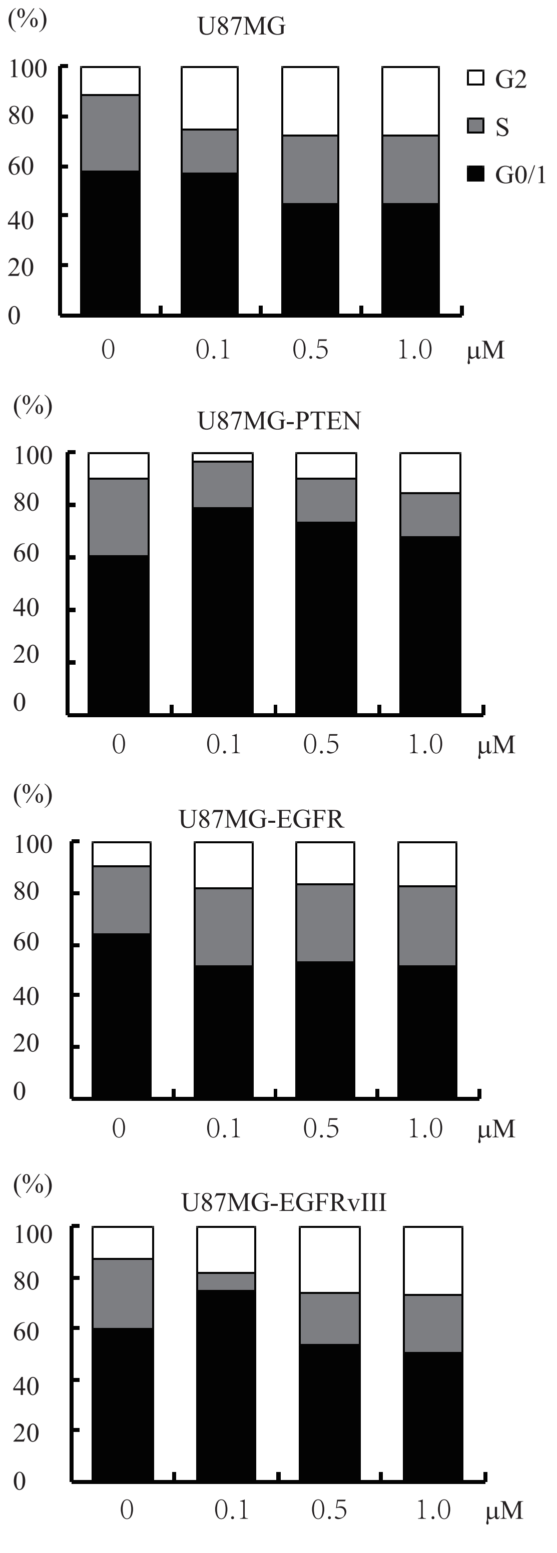
(%)
U87MG
100
80
60
40
20
0
0
0.1
0.5
1.0
μM
G2
S
G0/1
(%)
U87MG-PTEN
(%)
U87MG-EGFR
(%)
U87MG-EGFRvIII

B

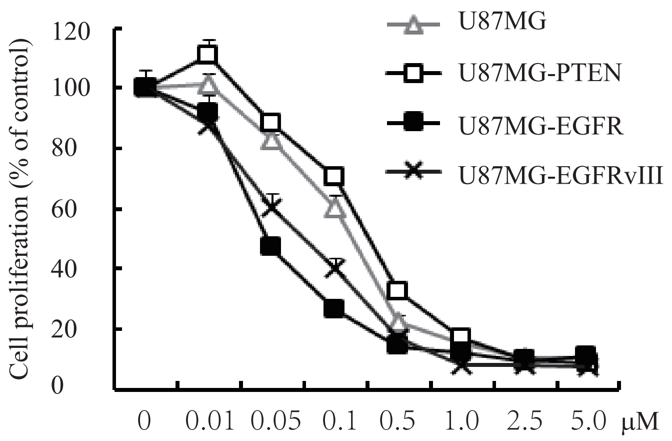
Cell proliferation (% of control)
120
100
80
60
40
20
0
0
0.01
0.05
0.1
0.5
1.0
2.5
5.0
μM
U87MG
U87MG-PTEN
U87MG-EGFR
U87MG-EGFRvIII

Figure S2

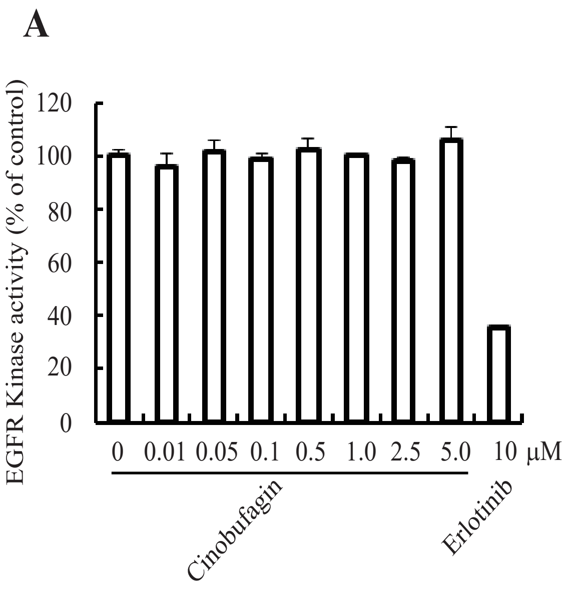
A
EGFR Kinase activity (% of control)
120
100
80
60
40
20
0
0
0.01
0.05
0.1
0.5
1.0
2.5
5.0
10
μM
Cinobufagin
Erlotinib

Figure S3

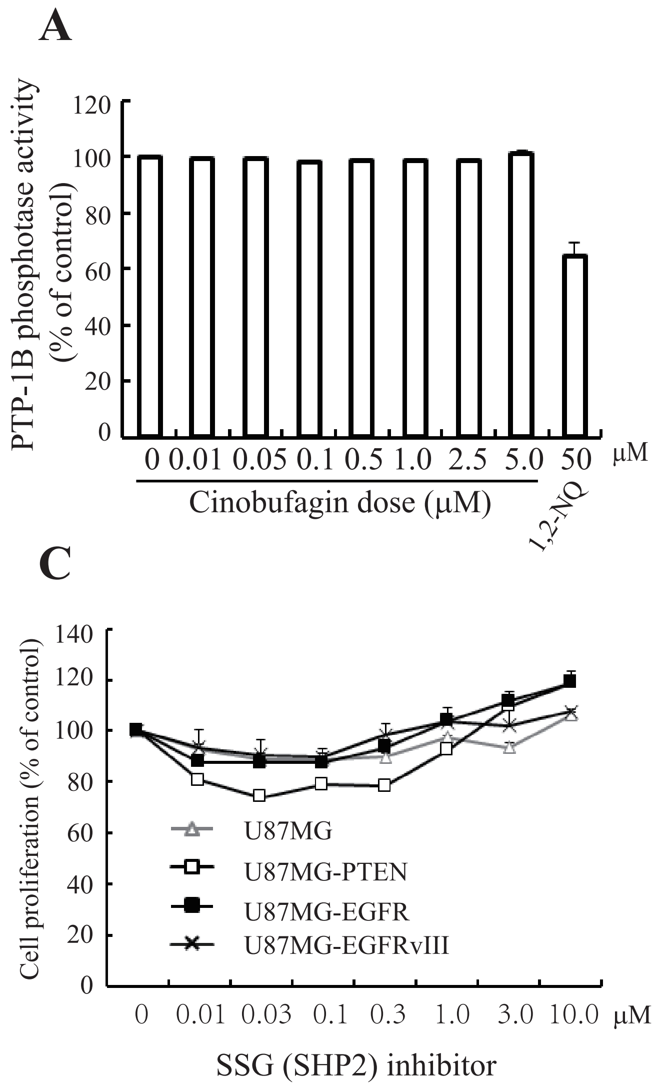
A
PTP-1B phosphotase activity (% of control)
120
100
80
60
40
20
0
0 0.01 0.05 0.1 0.5 1.0 2.5 5.0 50 μM
Cinobufagin dose (μM)
1,2-NQ
B
SHIP2 activity
Phosphate (pmol/well)
900
750
600
450
300
150
0
0 0.05 0.5 5.0
Cinobufagin dose (μM)
C
Cell proliferation (% of control)
140
120
100
80
60
40
20
0
U87MG
U87MG-PTEN
U87MG-EGFR
U87MG-EGFRvIII
0 0.01 0.03 0.1 0.3 1.0 3.0 10.0 μM
SSG (SHP2) inhibitor

Figure S4

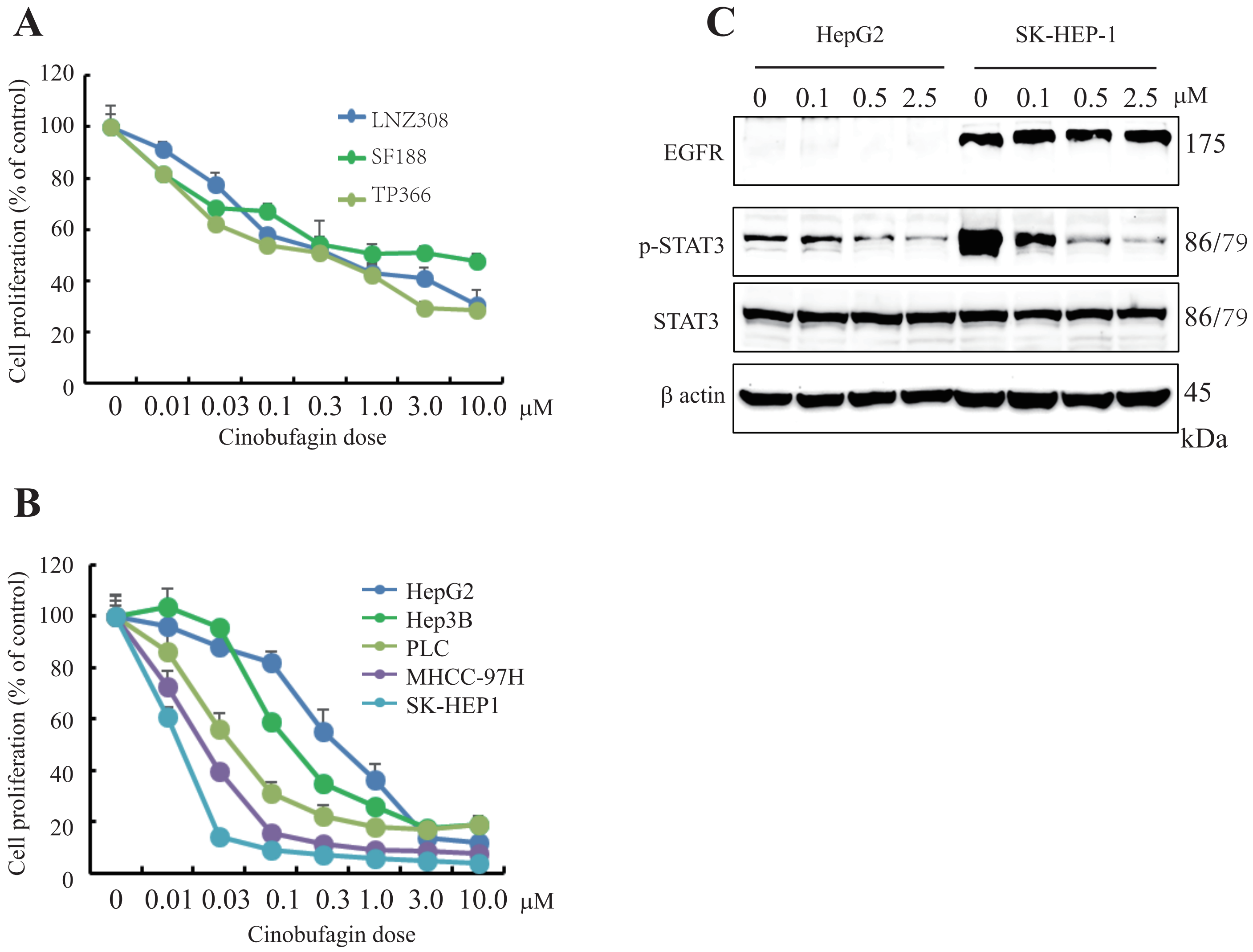
A
120
100
80
60
40
20
0
Cell proliferation (% of control)
LNZ308
SF188
TP366
0 0.01 0.03 0.1 0.3 1.0 3.0 10.0 μM
Cinobufagin dose
B
120
100
80
60
40
20
0
Cell proliferation (% of control)
HepG2
Hep3B
PLC
MHCC-97H
SK-HEP1
0 0.01 0.03 0.1 0.3 1.0 3.0 10.0 μM
Cinobufagin dose
C
HepG2
SK-HEP-1
0 0.1 0.5 2.5 0 0.1 0.5 2.5 μM
EGFR
175
p-STAT3
86/79
STAT3
86/79
β actin
45
kDa

Table S1. Kinase profiling of Cinobufagin. A panel of kinases was selected for the in vitro kinase profile screening (Invitrogen, SelectScreenTM Kinase Profiling). These assays employed the kinase domain recombinant proteins with 0.5 μM cinobufagin, 10 μM ATP or 100 μM ATP, if it is the available lowest concentration, and FRET (Fluorescence Resonance Energy Transfer)-peptide as substrates. The kinase assays were performed according to Invitrogen instructions.

Kinase Tested	% Inhibition	Kinase Tested	% Inhibition
BL1	-3	MINK1	-4
ADRBK1 (GRK2)	-1	MUSK	-2
AKT1 (PKB alpha)	13	NEK1	6
ALK	-1	NTRK1 (TRKA)	-1
AURKA (Aurora A)	2	NTRK2 (TRKB)	0
AXL	0	PAK1	1
CDC42 BPA (MRCKA)	-5	PDGFRA (PDGFR alpha)	-4
CDK1/cyclin B	0	PDK1	8
CDK5/p25	-2	PHKG1	-3
CHEK1 (CHK1)	3	PIM1	4
CLK1	4	PKN1 (PRK1)	9
CSF1R (FMS)	-3	PLK1	2
CSNK1A1 (CK1 alpha 1)	0	PRKACA (PKA)	-2
CSNK2A1 (CK2 alpha 1)	9	PRKCA (PKC alpha)	7
DNA-PK	9	PRKG1	5
DYRK1A	-2	PTK2 (FAK)	-10
EEF2K	-1	RET	5
EGFR (ErbB1)	-7	ROCK1	7
ERBB2 (HER2)	-11	RPS6KA1 (RSK1)	0
FER	6	RPS6KA5 (MSK1)	5
FGFR1	-9	RPS6KB1 (p70S6K)	-6
FGFR4	9	SGK (SGK1)	-2
FLT1 (VEGFR1)	-6	SRC	18
FRAP1 (mTOR)	1	STK4 (MST1)	1
FRK (PTK5)	-2	TEK (Tie2)	5
GRK4	-8	ZAP70	3
GSK3A (GSK3 alpha)	-3	CAMK1 (CaMK1)	-12
HCK	-9	DAPK1	-16
HIPK1 (Myak)	0	IRAK1	-11
IGF1R	3	LRRK2	-4
IKBKB (IKK beta)	0	PI4KA (PI4K alpha)	0
INSR	-3	PIK3C2A (PI3K-C2 alpha)	-4
JAK1	1	PIK3CA/PIK3R1 (p110 α/p85 α)	-12
JAK2	-3	PIK3CD/PIK3R1 (p110 δ/p85 α)	-13
KIT	2	PIK3CG (p110 gamma)	-14
LCK	0	AMPK (A1/B1/G2)	1
LTK (TYK1)	0	BRAF	2
MAPK14 (p38 alpha)	14	MAP2K1 (MEK1)	3
MAPK3 (ERK1)	3	MAP2K2 (MEK2)	-2
MAPK8 (JNK1)	3	RAF1 (cRAF) Y340D Y341D	6
MARK1 (MARK)	6	TGFBR1 (ALK5)	4
MET (cMet)	0		

Table S2. Phosphatase profiling of Cinobufagin. A panel of phosphatases was selected for the in vitro phosphatase profile screening (Eurofins Pharma Discovery Services UK Limited). These assays employed the phosphatase proteins with 1 μM cinobufagin. The phosphatase assays were performed according to Eurofins instructions.

Phosphatase tested	B8 @ 1 μM<br>Mean activity % of control
PTP-1B(h)	103
SHP-1(h)	94
SHP-2(h)	103
TCPTP(h)	98

Table S3. Complete blood count of vehicle and Cinobufagin treated mice.

C57BL/6J mice were orally treated with Cinobufagin for one month and the bloods were collected and analyzed.

Parameter	Vehicle	Cinobufagin
WBC ($\times 10^3/\mu l$)	5.56±1.14	3.75±0.88
Neutrophis ($\times 10^3/\mu l$)	1.0275±0.23	0.75±0.12
Neutrophis (% in WBC)	18.38±0.63	20.68±1.89
Lymphocytes ($\times 10^3/\mu l$)	4.25±0.83	2.83±0.76
Lymphocytes (% in WBC)	76.65±0.98	74.58±2.24
Monocytes ($\times 10^3/\mu l$)	0.07±0.06	0.02±0.00
Monocytes (% in WBC)	1.03±0.69	0.48±0.09
Eosinophils ($\times 10^3/\mu l$)	0.21±0.04	0.15±0.02
Eosinophils (% in WBC)	3.95±0.51	4.28±0.50
Basophils ($\times 10^3/\mu l$)	0.00±0.00	0.00±0.00
Basophils (% in WBCl)	0.00±0.00	0.00±0.00
RBC ($\times 10^6/\mu l$)	9.30±0.27	8.91±0.22
Hemoglobin (g/dl)	13.70±0.44	13.13±0.28
Hematocrit (%)	41.78±1.04	40.05±0.42
MCV (fl)	44.93±0.45	45.00±0.91
MCH (pg)	14.73±0.13	14.73±0.16
MCHC (g/dl)	32.78±0.28	32.80±0.67
Platelets ($\times 10^3/\mu l$)	1813.75±184.75	1605.75±286.07

Data are expressed as means ± S.E.M (n=4).
